# Use of Multiplex Molecular Panels to Diagnose Urinary Tract Infection in Older Adults

**DOI:** 10.1001/jamanetworkopen.2024.46842

**Published:** 2024-11-26

**Authors:** Kelly M. Hatfield, Sarah Kabbani, Isaac See, Dustin W. Currie, Christine Kim, Kara Jacobs Slifka, Shelley S. Magill, Lauri A. Hicks, L. Clifford McDonald, John Jernigan, Sujan C. Reddy, Joseph D. Lutgring

**Affiliations:** 1 Division of Healthcare Quality Promotion, Centers for Disease Control and Prevention, Atlanta, Georgia

## Abstract

**Importance:**

Multiplex molecular syndromic panels for diagnosis of urinary tract infection (UTI) lack clinical data supporting their use in routine clinical care. They also have the potential to exacerbate inappropriate antibiotic prescribing.

**Objective:**

To describe the frequency of unspecified multiplex testing in administrative claims with a primary diagnosis of UTI in the Medicare population over time, to assess costs, and to characterize the health care professionals (eg, clinicians, laboratories, physician assistants, and nurse practitioners) and patient populations using these tests.

**Design, Setting, and Participants:**

This cohort study used Centers for Medicare & Medicaid Services (CMS) claims data for Medicare beneficiaries. The study included older community-dwelling adults and nursing home residents with fee-for-service Medicare Part A and Part B benefits from January 1, 2016, to December 31, 2023.

**Main Outcomes and Measures:**

Multiplex syndromic panels were identified using carrier claims (ie, claims for clinician office or laboratory services). The annual rate of claims was measured for multiplex syndromic panels with a primary diagnosis of UTI per 10 000 eligible Medicare beneficiaries. The performing and referring specialties of health care professionals listed on claims of interest and the proportion of claims that occurred among beneficiaries residing in a nursing home were described.

**Results:**

Between 31 110 656 and 36 175 559 Medicare beneficiaries with fee-for-service coverage annually (2016-2023) were included in this study. In this period, 1 679 328 claims for UTI multiplex testing were identified. The median age of beneficiaries was 77 (IQR, 70-84) years; 34% of claims were from male beneficiaries and 66% were from female beneficiaries. From 2016 to 2023, the observed rate of UTI multiplex testing increased from 2.4 to 148.1 claims per 10 000 fee-for-service beneficiaries annually, and the proportion of claims that occurred among beneficiaries residing in a nursing home ranged from 1% in 2016 to 12% in 2020. In addition to laboratories or pathologists, urology was the most common clinician specialty conducting this testing. The CMS-assigned referring clinician specialty was most frequently urology or advanced practice clinician for claims among community-dwelling beneficiaries compared with internal medicine or family medicine for claims among nursing home residents. In 2023, the median cost of a multiplex test in the US was $585 (IQR, $516-$695 for Q1-Q3), which was more than 70 times higher than the median cost of $8 for a urine culture (IQR, $8-$16 for Q1-Q3).

**Conclusions and Relevance:**

This cohort study of Medicare beneficiaries with fee-for-service coverage from 2016 to 2023 found increasing use of emerging multiplex testing for UTI coupled with high costs to the Medicare program. Monitoring and research are needed to determine the effects of multiplex testing on antimicrobial use and whether there are clinical situations in which this testing may benefit patients.

## Key Points

**Question:**

How frequently are multiplex molecular syndromic panels used to diagnose urinary tract infection (UTI) among older Medicare beneficiaries?

**Findings:**

In this cohort study of older adults, Medicare fee-for-service claims for multiplex testing with a primary diagnosis of UTI increased from 2 to 148 claims per 10 000 beneficiaries annually between 2016 and 2023.

**Meaning:**

These findings suggest that claims for costly multiplex molecular testing for UTI are frequent among Medicare beneficiaries, including nursing home residents, despite a lack of clinical evidence supporting their value.

## Teaser

This cohort study uses Medicare fee-for-service claims data to assess the use of multiplex molecular panels to diagnose urinary tract infection in older adults from 2016 to 2023.

## Introduction

Clinical microbiology advances in the past decade have resulted in several new technologies to diagnose infectious diseases.^1,2^ Multiplex molecular syndromic panels are rapid diagnostic assays that can simultaneously detect multiple pathogens and some antimicrobial resistance genes, and they have changed how infectious diseases are diagnosed.^3^ US Food and Drug Administration (FDA)-approved panels are now used in the care of patients with bloodstream, respiratory, gastrointestinal, central nervous system, and sexually transmitted infections.^4,5,6,7,8,9^ Benefits of this testing include quicker time to results, increased sensitivity, and improved laboratory workflow.^2,5,6,8,9^ Some panels have been demonstrated to improve antimicrobial use and patient outcomes.^5,6,8,9^ Limitations associated with these tests include decreased clinical specificity, difficulty in interpreting the results when pathogens on the panel have differing pretest probabilities of disease, and cost.^3,10^

There are currently no FDA-approved multiplex molecular syndromic panels to diagnose urinary tract infection (UTI), but such panels exist as laboratory-developed tests (LDTs).^4,11,12,13,14,15^ Organism detection rates of multiplex molecular panels have been compared to urine culture, but data demonstrating benefit to patients compared with culture (eg, improved symptom relief, cure rates, or more appropriate antibiotic use) are lacking.^11,12,13,14,15^ LDTs can be used in a laboratory that is certified under the Clinical Laboratory Improvement Amendments of 1988 (CLIA) and meets the CLIA regulatory requirements of high-complexity testing. Due to concerns regarding the safety and effectiveness of these tests, the FDA announced in 2024 plans to increase the regulation of LDTs.^16^ Multiplex panels for UTIs are of particular interest for monitoring, given the large number of UTIs and the frequency with which antibiotics are given for asymptomatic bacteriuria, which may result in patient harm.^17,18^

To date, the uptake and use of laboratory-developed multiplex molecular syndromic panels for diagnosing UTI has not been well described. Monitoring the use of laboratory-developed assays is challenging using claims-based data sources, because most tests do not have specific *Current Procedural Terminology, Fourth Edition* (*CPT-4*) codes. Although laboratory-developed assays sometimes have Proprietary Laboratory Analyses (PLA) codes in the *CPT* set that allow for laboratories or manufacturers to specifically identify their tests, we did not identify any PLA or *CPT-4* codes specific to multiplex testing for UTI. Thus, the objective of this analysis was to systematically identify multiplex testing for UTI in administrative claims data to describe the frequency of its use in the Medicare population over time. Furthermore, we aimed to characterize the health care professionals (eg, clinicians, laboratories, physician assistants, and nurse practitioners) and patient populations using multiplex testing and to assess the costs of these tests.

## Methods

This cohort study was conducted under a data use agreement with the Centers for Medicare & Medicaid Services (CMS). This activity was reviewed by the Centers for Disease Control and Prevention (CDC), was deemed not human participant research with a waiver of informed consent, and was conducted consistent with applicable federal law and CDC policy (eg, 45 CFR §46, 21 CFR §56, 42 USC §241(d), 5 USC §552a, and 44 USC 277 §3501 et seq). The study followed the Strengthening the Reporting of Observational Studies in Epidemiology (STROBE) reporting guideline.

### Data Sources

We assessed the number and rate of paid claims for UTI multiplex tests in CMS data. We used data from the Medicare beneficiary summary files, Minimum Dataset 3.0 (MDS) assessment files, and Part B carrier claims in the Chronic Conditions Warehouse Virtual Research Data Center between January 1, 2016, and December 31, 2023. To ascertain an annual denominator for the number of eligible fee-for-service beneficiaries, we counted the total number of Medicare beneficiaries each year who had fee-for-service Part A and Part B benefits for any month in the year. We also created an annual denominator for the number of unique fee-for-service beneficiaries who resided at least 1 day in a nursing home in the specified year by identifying a subset of the beneficiaries (per the aforementioned description) who had at least 1 MDS assessment in the annual files. MDS assessments are a required health status screening and assessment tool used for all residents of long-term care nursing facilities certified by CMS at admission and regular intervals (with no less than 1 assessment every 3 months).

### Outcomes

We developed an algorithm to identify Medicare Part B carrier claims (ie, claims for clinician office or laboratory services) for unspecified multiplex testing. Part B carrier claims include overall claim information (eg, beneficiary information, service dates, claim types, diagnosis codes, and overall payment amounts) and 1 or more detailed line items, which include specific information regarding a specific procedure, supply, product, or service rendered.^19^ First, we identified claims with *CPT-4* codes indicating the detection of an infectious agent using a nucleic acid probe (eTable 1 in Supplement 1). Our algorithm required claims to have at least 3 procedure codes of interest (ie, a *CPT-4* code indicating detection of an infectious agent using a nucleic acid probe), with at least 1 *CPT-4* code for nucleic acid detection of multiple organisms or a nonspecified organism (eTable 1 in Supplement 1). We excluded any carrier claim with a line item that included a *CPT-4* procedure code for a multiplex panel specific to another infection type (ie, panels targeting ≥3 pathogens specific to a respiratory or pneumonia infection, urogenital or anogenital infection, bloodstream infection, central nervous system infection, or gastrointestinal infection) (eTable 2 in Supplement 1). Denied claims (ie, claims with an overall payment of $0 from CMS) were also excluded in our analysis.

Claims identified were considered indicative of multiplex testing but with an unspecified infection type (ie, unspecified multiplex claims) and categorized based on the primary *International Classification of Diseases, Tenth Revision, Clinical Modification* (*ICD-10-CM*) diagnosis code of the claim as a urinary tract, urogenital or anogenital, respiratory, nail and skin or soft tissue, or gastrointestinal infection (eTable 3 in Supplement 1 includes a complete list of *ICD-10-CM* diagnosis codes). We calculated the rate of unspecified multiplex claims with a primary diagnosis categorized as UTI (ie, UTI multiplex claims) per 10 000 Medicare fee-for-service beneficiaries each year. We assessed the CMS Performing Provider Specialty code listed on the associated line items of interest. These codes are assigned by Medicare using the clinician’s type, classification, or specialization selected when applying for their National Provider Identifier specialty and may include a clinician specialty grouping, advanced practice clinician categorization (ie, physician assistant or nurse practitioner), or designation of clinical laboratory.^20^ For UTI multiplex claims, laboratories conducting this testing were identified by the CLIA laboratory number listed on the associated line items. Finally, for all unspecified multiplex claims with a UTI primary diagnosis, we summed individual line payment amounts for all nucleic acid test lines of interest to determine the multiplex test cost for each claim.

To assess the occurrence of UTI multiplex testing among nursing home residents, we used admission and discharge dates listed on the MDS assessments to determine whether a beneficiary was residing in a nursing home on the date of the UTI multiplex claim. If a beneficiary had an assessment date within 90 days of a UTI multiplex claim that did not have a discharge date listed, we included them as a nursing home resident.

### Statistical Analysis

We calculated the annual rate of multiplex claims with a primary diagnosis of UTI among beneficiaries residing in a nursing home at the time of testing per 10 000 Medicare fee-for-service beneficiaries who resided in a nursing home at any point in the year of interest. We compared the specialty codes for referring health care professionals for the overall claim for beneficiaries who were residing in the community with those for beneficiaries who were residing in a nursing home at the time of their claim.

For comparison, we identified carrier claims for urine cultures (ie, claims with a line item containing *CPT-4* code 87086 or 87088, specifying urine culture) with a primary diagnosis categorized as UTI (eTable 3 in Supplement 1). We identified health care professional specialty type, nursing home residence, cost of line items, and rate of claims for urine cultures using the same approach as multiplex testing. All analyses were conducted using SAS Studio, release 3.82 (SAS Institute).

## Results

**Figure 1.**
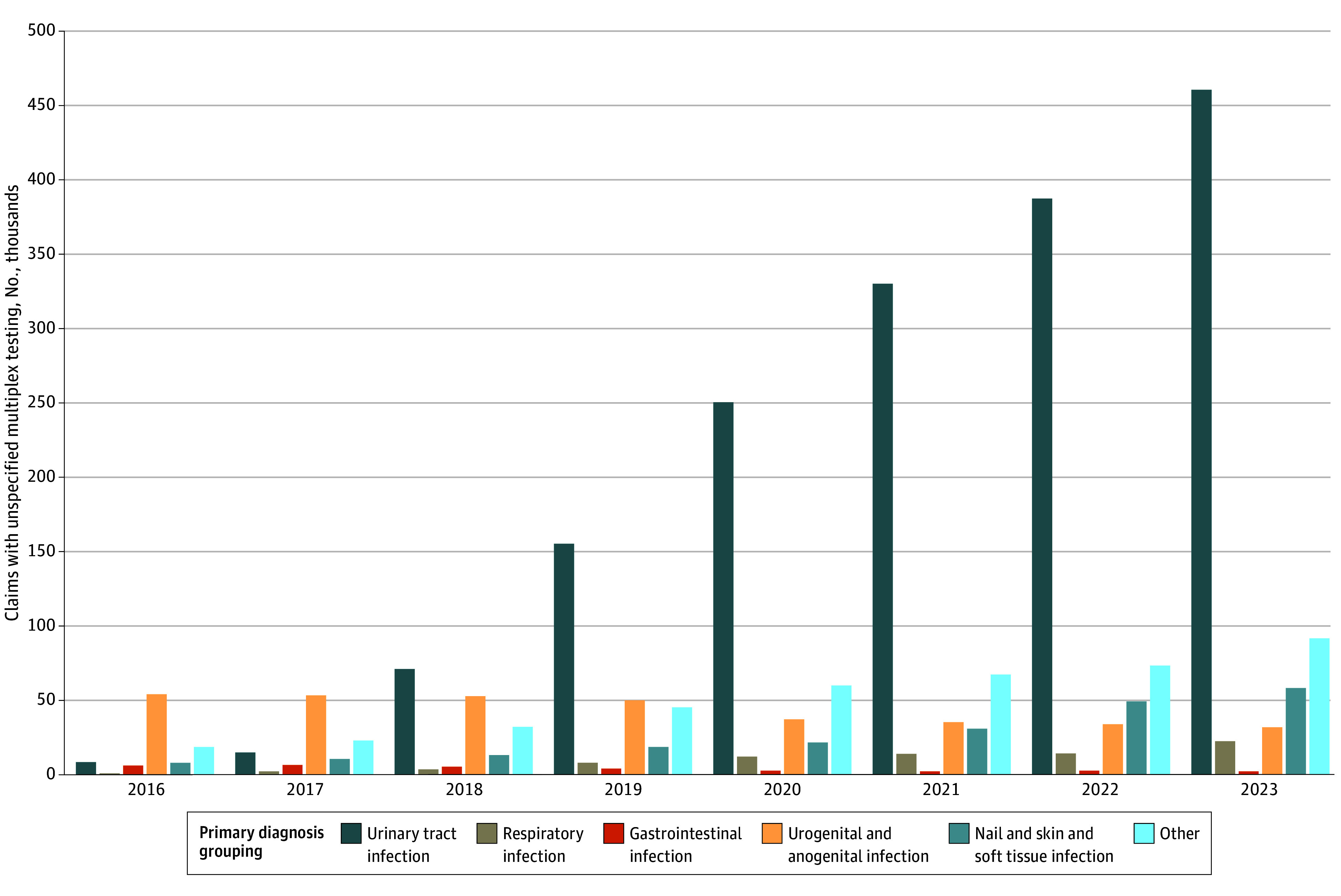
Annual Number of Carrier Claims With Procedure Codes Indicating Unspecified Multiplex Tests Stratified by Primary Infection Diagnosis, 2016-2023 *Current Procedural Terminology, Fourth Edition* procedure codes were used. Data are from the Centers for Medicare & Medicaid Services Chronic Conditions Warehouse.

For 2016 to 2023, we identified between 31 110 656 and 36 175 559 beneficiaries with traditional fee-for-service (ie, Part A and Part B) coverage annually (Table 1). Among these beneficiaries, 4% to 7% had a nursing home stay identified by an MDS assessment in that year. Using Part B Medicare claims, we identified 2 934 748 claims for unspecified multiplex testing, of which 1 679 328 (57%) had a primary diagnosis categorized as UTI. The median age of beneficiaries with these claims was 77 (IQR, 70-84 for quartiles 1-3 [Q1-Q3]) years; 34% of these claims were from male beneficiaries and 66% were from female beneficiaries. Annual claims for unspecified multiplex molecular tests increased from 98 817 in 2016 to 710 378 in 2023, attributable to increases in claims with a primary diagnosis of UTI (Figure 1).

**Table 1.** Characteristics of Medicare Beneficiaries and Carrier (Noninstitutional) Claims With Urine Cultures and Unspecified Multiplex Testing With the Primary Diagnosis Identified as UTI, 2016 to 2023^a^

Characteristic	Year							
	2016 (n = 36 175 559)	2017 (n = 36 108 243)	2018 (n = 35 998 687)	2019 (n = 35 717 485)	2020 (n = 34 897 583)	2021 (n = 33 540 244)	2022 (n = 32 265 949)	2023 (n = 31 110 656)
All FFS beneficiaries with an MDS assessment in that year^b^	2 540 641 (7)	2 471 202 (7)	2 385 764 (7)	2 292 231 (6)	1 863 969 (5)	1 772 392 (5)	1 761 697 (5)	1 395 832 (4)
No. of Part B UTI claims								
With urine culture	4 249 139	4 207 744	4 120 640	4 099 727	3 364 006	3 453 580	3 437 548	3 472 523
With multiplex testing	8521	15 031	71 158	155 384	250 629	330 282	387 617	460 706
Beneficiaries with multiplex claims								
Age at time of claim, median (IQR), y	69 (60-76)	73 (67-80)	75 (69-83)	75 (68-83)	77 (70-84)	77 (70-84)	77 (70-84)	77 (71-85)
Sex								
Male	1151 (14)	3571 (24)	23 920 (34)	52 371 (34)	87 869 (35)	121 665 (37)	134 717 (35)	153 083 (33)
Female	7370 (86)	11 460 (76)	47 238 (66)	103 013 (66)	162 760 (65)	208 617 (63)	252 900 (65)	307 623 (67)
Nursing home resident	61 (1)	216 (1)	4959 (7)	7929 (5)	30 097 (12)	20 993 (6)	27 735 (7)	34 603 (8)
Claim payment (sum of multiplex-related items), median (IQR), $	206 (140-656)	347 (150-708)	453 (235-637)	573 (284-726)	561 (413-702)	597 (515-772)	597 (482-702)	585 (516-695)
Specialty of health care professional performing associated tests^c^								
Laboratory or pathology	8516 (>99)	15 005 (>99)	68167 (96)	142 602 (92)	206 613 (82)	241 426 (73)	304 340 (79)	395 104 (86)
Urology	0	0	2639 (4)	11 483 (7)	40 068 (16)	79 151 (24)	71 274 (18)	52 630 (11)
Advanced practice clinician	0	<11 (<1)	76 (<1)	747 (<5)	2121 (1)	5753 (2)	7214 (2)	9068 (2)
Obstetrics and gynecology	<11 (<0.1)	<11 (<1)	58 (<1)	82 (1)	658 (<1)	1856 (1)	1753 (1)	790 (<1)
Internal medicine	0	<11 (<1)	60 (<1)	310 (<1)	457 (<1)	955 (<1)	1312 (<1)	1302 (<1)
Family medicine	0	<11 (<1)	74 (<1)	68 (<1)	511 (<1)	963 (<1)	1218 (<1)	604 (<1)
Other	<11 (<1)	<11 (<1)	84 (<1)	92 (<1)	201 (<1)	178 (<1)	506 (<1)	1208 (<1)

Abbreviations: FFS, fee-for-service; MDS, Minimum Dataset 3.0; UTI, urinary tract infection.

^a^ Data are from the Centers for Medicare & Medicaid Services Chronic Conditions Warehouse and are expressed as the No. (%) of beneficiaries or claims unless indicated otherwise.

^b^ Percentages in this row are with respect to the total number of patients covered by Medicare in a given year.

^c^ Includes clinicians, laboratories, physician assistants, and nurse practitioners.

**Figure 2.**
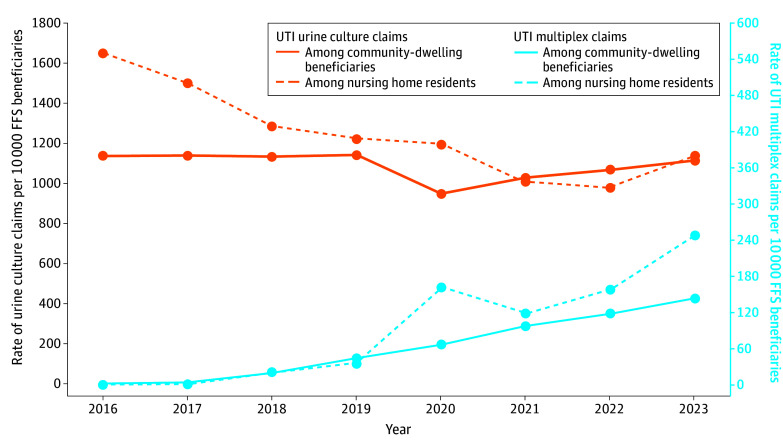
Annual Rate of Claims Per 10 000 Fee-for-Service (FFS) Medicare Beneficiaries With a Primary Diagnosis of Urinary Tract Infection (UTI) and Procedure Codes Indicating Urine Culture and Multiplex Testing, 2016-2023 *Current Procedural Terminology, Fourth Edition* diagnosis codes were used. Claims that occurred while a beneficiary was residing in a nursing home are presented per 10 000 fee-for-service beneficiaries with at least 1 Minimum Dataset 3.0 assessment each year. Data are from the Centers for Medicare & Medicaid Services Chronic Conditions Warehouse.

From 2016 to 2023, the observed rate of UTI multiplex testing increased from 2.4 to 148.1 claims per 10 000 fee-for-service beneficiaries (Figure 2). This rate remained substantially lower than the rate of 1116.2 claims per 10 000 fee-for-service beneficiaries for urine cultures with a primary diagnosis of UTI in 2023; however, the rate for urine cultures did not increase during the same period (Figure 2). The rate of UTI multiplex claims among beneficiaries residing in a nursing home increased from 0.24 to 247.9 per 10 000 fee-for-service beneficiaries from 2016 to 2023; the rate of UTI multiplex testing among nursing home residents increased more than the rate of UTI multiplex claims among community-dwelling Medicare beneficiaries beginning in 2020 (Figure 2). In comparison, the rate of claims with urine cultures and a primary diagnosis of UTI was higher among beneficiaries residing in a nursing home compared with community-dwelling Medicare beneficiaries from 2016 to 2020, lower from 2021 to 2022, and slightly higher in 2023.

In 2023, there were 460 706 claims for UTI multiplex tests. Most claims (307 623 [67%]) were among female beneficiaries, and the median age of beneficiaries with claims was 77 (IQR, 71-85 for Q1-Q3) years (Table 1). In 2023, the median cost for line items of interest on UTI multiplex claims was $585 (IQR, $516-$695 for Q1-Q3) per claim (Table 1) compared with $8 (IQR, $8-$16 for Q1-Q3) per claim for corresponding line items on UTI urine culture claims.

In all years, most line items of interest were performed by clinician specialty types classified as from laboratories or pathologists (Table 1). Throughout the study period, UTI multiplex claims were associated with more than 900 laboratories, and 45 states had at least 10 claims. The next most common clinician specialty listed was urology (the performing clinician on 4% of claims in 2018 and 11% of claims in 2023, with a high of 24% of claims in 2021) (Table 1).

Among all claims for UTI multiplex testing, the proportion of beneficiaries who were residing in a nursing home at the time of the claim ranged from 1% in 2016 to 12% in 2020. Notably, there were differences in the referring clinician specialty coded listed on claims for nursing home residents compared with those on claims for community-dwelling beneficiaries. Community-dwelling beneficiaries were most likely to have a referring clinician with a specialty of urology (32%) or a referring advanced practice clinician (28%), whereas nursing home residents were more likely to have a referring clinician with a family medicine (39%) or internal medicine (30%) specialty (Table 2).

**Table 2.** Distribution of Clinician Specialty for Referring Clinicians Listed on Medicare Claims With Unspecified Multiplex Testing and a Primary Diagnosis of UTI^a^

Specialty of referring clinician	Medicare Part B claims with multiplex testing and a primary diagnosis of UTI	
	Community-dwelling beneficiaries (n = 1 552 735)	Nursing home residents (n = 126 593)
Urology	493 977 (32)	5812 (5)
Advanced practice clinician^b^	433 081 (28)	18 782 (15)
Internal medicine	197 801 (13)	37 848 (30)
Family medicine	164 718 (11)	49 989 (39)
Other	207 072 (13)	11 562 (9)
Unknown	56 086 (4)	2600 (2)

Abbreviation: UTI, urinary tract infection.

^a^ Data are from the Centers for Medicare & Medicaid Services Chronic Conditions Warehouse and are expressed as the No. (%) of claims.

^b^ Includes nurse practitioners and physician assistants.

## Discussion

In this large retrospective study of fee-for-service Medicare claims, the use of multiplex testing to diagnose UTI in older adults in the US increased more than 60-fold (from 2.4 to 148.1 claims per 10 000 beneficiaries annually) between 2016 and 2023. Notably, multiplex testing to diagnose UTI among nursing home residents occurred at a higher rate than that among community-dwelling beneficiaries beginning in 2020. Outside of laboratories and pathologists, urology was the most common clinician specialty performing these tests and was the most common clinician specialty referring community-dwelling patients for these tests. We also found that in 2023, the median cost per claim for multiplex testing with a primary diagnosis of UTI was nearly 70 times higher than the median cost of urine culture claims with a UTI primary diagnosis ($585 vs $8, respectively).

UTI is a common and leading reason for outpatient antibiotic use, particularly among older adults.^17,18^ Urine culture is the traditional benchmark for diagnosis of UTI; however, organisms can be cultured from urine specimens in the absence of clinical infection (ie, asymptomatic bacteriuria), and results of urine cultures may not be available immediately. UTI is a clinical diagnosis, and accurately differentiating asymptomatic bacteriuria from a UTI that needs antibiotic treatment is often challenging among older adults. However, failure to appropriately diagnose UTI can lead to unnecessary antibiotic use and diagnostic errors.^21^ Treatment of asymptomatic bacteriuria may lead to adverse outcomes such as the potential for colonization or infection with antimicrobial-resistant pathogens, adverse drug events, and *Clostridioides difficile* infection.^22^ As a result, there is a need for innovative diagnostics to improve turnaround times and to better differentiate asymptomatic bacteriuria from UTI.^23^

Multiplex testing generates faster results and is more analytically sensitive than traditional urine culture; a recent review showed that between 7% and 40% of negative urine cultures were polymerase chain reaction positive.^11^ However, this increased analytical sensitivity may lead to increased detection of asymptomatic bacteriuria and decreased specificity for the diagnosis of UTI. For example, in a study comparing urine cultures with nucleic acid detection, 5 of 22 asymptomatic control participants (23%) had a positive urine culture and 21 (95%) had a positive nucleic acid next-generation sequencing test result.^24^ There is currently insufficient evidence to compare patient responses to antibiotic therapy when diagnosed using molecular tests vs standard urine culture methods, and there is a lack of literature showing clinical populations that would benefit from this testing or outcomes that demonstrate their utility in routine practice.^11^ Furthermore, some of these tests detect genetic information for numerous organisms and the presence of associated resistance genes simultaneously. If it is not known which organism harbors which resistance gene, it may be difficult to determine how to use this information to care for patients.^23^

Nursing home residents appear to increasingly receive multiplex testing to diagnose UTI despite limited evidence for its utility in this setting.^15^ Nursing home residents are frequently inappropriately treated for asymptomatic bacteriuria because of additional challenges distinguishing colonization or contamination from infection in this population.^25,26,27^ Because of the high rate of inappropriate antimicrobial use occurring in nursing homes, additional scrutiny of urine multiplex testing is needed in this patient population.^15,28,29,30^ Detection of asymptomatic bacteriuria and genitourinary bacterial colonization that is not causing disease might be more frequent with urine multiplex than culture, potentially increasing unnecessary antibiotic use and harm to patients.^24^

In this study, different types of clinicians referred community-dwelling and nursing home patients for multiplex testing; therefore, multiple specialties and audiences should be made aware of the lack of data supporting the use of this testing.^15^ Education about these limitations should focus on those caring for nursing home patients. In 2024, the FDA announced that there will be increased oversight of LDTs, such as urine multiplex testing, which may affect the use of these tests in the future.^16^

### Limitations

This study has several limitations. First, because there is no specific procedure code for identifying UTI multiplex tests, we developed an algorithm for identifying these tests in claims. This algorithmic approach has the potential for misidentifying claims as unspecified multiplex tests that did not have this type of testing. Additionally, this algorithm relied on the listed primary diagnosis code to characterize multiplex tests used for diagnosis of UTIs. This method may undercount UTI multiplex tests if a different primary diagnosis was provided. Second, we limited our analysis to paid fee-for-service claims. We were unable to describe the burden of this type of testing in the Medicare population that was denied payment or self-paid by individuals, meaning our estimates would not capture the true occurrence of this testing among older adults. Third, we were limited in our classification of performing health care professional specialty using Medicare-assigned categorization in the claims data. Clinical specialties were limited to clinicians, and the specialties of advanced practice clinicians (ie, nurse practitioners and physician assistants) were not able to be ascertained. Finally, our analysis was limited to fee-for-service claims and did not include data from the Medicare Advantage population. Because Medicare Advantage programs are incentivized to use capitation in the cost of care, it is possible that they have more stringent requirements to justify payment for this type of testing.

## Conclusions

This cohort study identified important increases in the use of multiplex molecular syndromic panels for UTI, coupled with high costs to CMS, since 2016. Clinicians should be aware of the lack of data supporting this testing and the potential to further contribute to inappropriate antibiotic prescribing. We found recent, dramatic increases in the use of multiplex molecular tests for UTI as well as the substantial costs associated with these tests, despite a lack of evidence supporting their value for patient care and the significant potential for inappropriate antibiotic prescribing.^15^ Clinicians and payers may also consider the paucity of data demonstrating the benefit of this testing compared with the standard practice (eg, urine culture). In the absence of these data, increased FDA oversight of newly developed LDTs may affect the use of multiplex testing for UTI. Additional monitoring and research are needed to determine the effects of multiplex testing to diagnose UTI on antimicrobial use and whether there are clinical situations in which this testing may benefit patients.

## Notes

Supplement 1. **eTable 1.** *CPT-4* Procedure Codes for Tests That Were Counted as Line Items of Interest Using Nucleic Acid Detection Testing **eTable 2.** *CPT-4* Codes for Multiplex Tests Used to Diagnose an Infection Other Than a Urinary Tract Infection **eTable 3.** Categorizations of Primary Diagnosis by *ICD-10-CM* Diagnosis Codes

Supplement 2.
Data Sharing Statement
