# Supplementary material for: Use of Multiplex Molecular Panels to Diagnose Urinary Tract Infection in Older Adults
Source: JAMA Netw Open. 2024 Nov 26;7(11):e2446842. doi: 10.1001/jamanetworkopen.2024.46842 (PMC11600226; doi:10.1001/jamanetworkopen.2024.46842)
Supplement: Supplement 1. — eTable 1. CPT-4 Procedure Codes for Tests That Were Counted as Line Items of Interest Using Nucleic Acid Detection Testing eTable 2. CPT-4 Codes for Multiplex Tests Used to Diagnose an Infection Other Than a Urinary Tract Infection eTable 3. Categorizations of Primary Diagnosis by ICD-10-CM Diagnosis Codes [file jamanetwopen-e2446842-s001.pdf]

Supplementary Online Content

Hatfield KM, Kabbani S, See I, et al. Use of multiplex molecular panels to diagnose urinary tract infection in older adults. *JAMA Netw Open*. 2024;7(11):e2446842. doi:10.1001/jamanetworkopen.2024.46842

eTable 1. *CPT-4* Procedure Codes for Tests That Were Counted as Line Items of Interest Using Nucleic Acid Detection Testing

eTable 2. *CPT-4* Codes for Multiplex Tests Used to Diagnose an Infection Other Than a Urinary Tract Infection

eTable 3. Categorizations of Primary Diagnosis by *ICD-10-CM* Diagnosis Codes

This supplementary material has been provided by the authors to give readers additional information about their work.

© 2024 Hatfield KM et al. *JAMA Network Open.*

eTable 1. *CPT-4* Procedure Codes for Tests That Were Counted as Line Items of Interest Using Nucleic Acid Detection Testing

Claims were required to have at least 3 distinct lines of interest with the below listed CPT-4 codes to be considered an unspecified multiplex claim.

CPT-4 Code	Translation	Pathogen Type
87480	test for Candida species using direct nucleic acid probe technique.	Specific
87481	Candida species using amplified nucleic acid probe technique.	Specific
87640	The lab analyst performs the technical lab test to look for Staphylococcus aureus nucleic acid using an amplified probe technique.	Specific
87641	test for methicillin–resistant Staphylococcus aureus (MRSA) using an amplified nucleic acid probe technique	Specific
87651	test for the bacteria Streptococcus, group A, using amplified nucleic acid probe technique	Specific
87653	test for Streptococcus, group B, using amplified nucleic acid probe technique. The amplified probe provides a rapid means to assess colonization by Streptococcus, group B, as in the vaginal tracts of pregnant women	Specific
87797	test for an infectious agent, not otherwise specified in another code, in the specimen using direct nucleic acid probe technique	Non-specified
87798	test for an infectious agent not specified in another code using an amplified nucleic acid probe technique	Non-specified
87799	test to quantify infection with an infectious agent, not otherwise specified in another code, using a nucleic acid probe technique	Non-specified
87800	test for multiple infectious organisms in one sample using direct nucleic acid probe technique	Non-specified, Multiple
87801	a test for multiple infectious organisms in one sample using amplified nucleic acid probe technique	Non-specified, Multiple

Note: *Current Procedural Terminology*® (CPT), Fourth Edition (CPT-4). Line items of interest were not required to be distinct (i.e., a claim could be considered for inclusion if it has three individual lines with CPT-4 code 87798, which indicates testing for an infectious agent not specified in another code using an amplified nucleic acid probe technique).

© 2024 Hatfield KM et al. *JAMA Network Open.*

eTable 2. *CPT-4* Codes for Multiplex Tests Used to Diagnose an Infection Other Than a Urinary Tract Infection

Claims with a line item for any of these tests were excluded.

CPT-4 Code	Short Description	Panel Targeted Infection Type
81513	nucleic acid test on a vaginal fluid specimen for 3 bacterial species for bacterial vaginosis	Urogenital/Anogenital
81514	nucleic acid test on a vaginal fluid specimen for 6 bacterial species for bacterial vaginosis	Urogenital/Anogenital
87154	nucleic acid test on a blood specimen for 6 or more bacterial species	Bloodstream
87483	multiplex nucleic acid test to identify central nervous system pathogens	Central nervous system
87505	nucleic acid test to detect 3 to 5 types of gastrointestinal infection pathogens	Gastrointestinal
87506	nucleic acid test to detect 6 to 11 types of gastrointestinal infection pathogens	Gastrointestinal
87507	nucleic acid test to detect 12 to 25 types of gastrointestinal infection pathogens	Gastrointestinal
87631	nucleic acid test to detect 3 to 5 types of pulmonary/respiratory infection pathogens	Pulmonary/Respiratory
87632	nucleic acid test to detect 6 to 11 types of pulmonary/respiratory infection pathogens	Pulmonary/Respiratory
87633	nucleic acid test to detect 12 to 25 types of pulmonary/respiratory infection pathogens	Pulmonary/Respiratory
87636	nucleic acid test to detect SARS-CoV-2, influenza A, and influenza B	Pulmonary/Respiratory
87637	nucleic acid test to detect SARS-CoV-2, influenza A, influenza B, and RSV	Pulmonary/Respiratory
0115U	Proprietary Laboratory Analyses (PLA): Respiratory Panel	Pulmonary/Respiratory
0202U	Proprietary Laboratory Analyses (PLA): Respiratory Panel	Pulmonary/Respiratory
0223U	Proprietary Laboratory Analyses (PLA): Respiratory Panel	Pulmonary/Respiratory
0225U	Proprietary Laboratory Analyses (PLA): Respiratory Panel	Pulmonary/Respiratory
0240U	Proprietary Laboratory Analyses (PLA): Respiratory Panel	Pulmonary/Respiratory
0241U	Proprietary Laboratory Analyses (PLA): Respiratory Panel	Pulmonary/Respiratory
0352U	Proprietary Laboratory Analyses (PLA): Vaginal Panel	Urogenital/Anogenital
0353U	Proprietary Laboratory Analyses (PLA): Sexually Transmitted Infection Panel	Urogenital/Anogenital

Note: *Current Procedural Terminology*® (CPT), Fourth Edition (CPT-4).

eTable 3. Categorizations of Primary Diagnosis by *ICD-10-CM* Diagnosis Codes

	**International Classification of Diseases, Tenth Revision Clinical Modification (ICD-10-CM) diagnosis codes**
Gastrointestinal	A000, A009, A020, A039, A044, A045, A047, A0471, A0472, A048, A049, A071, A073, A078, A080, A0811, A0839, A084, A088, A09, A681, B820, B829, K520, K521, K522, K5229, K523, K52831, K52832, K52839, K5289, K529, K580, K582, K591, K6389, K921, K929, R1110, R1111, R112, R152, R159, R195, R197, Z110, Z2009
Nail, Skin and Soft Tissue Infections	A260, A311, B001, B028, B029, B070, B350B351 B352 B353, B354, B355, B356, B358, B359, B360, B362, B368, B369, B372, B86, B871, E08621, E08622, E10621, E10622, E1152, E11621, E11622, E13621, E13622, H6000, H6001, H6002, H6011, H6012, I70232, I70233, I70234, I70235, I70238, I70242, I70243, I70244, I70245, I70248, I7025, I70261, I83009, I83013, I83015, I83018, I83023, I83025, I83028, I83029, I83212, I83213, I83222, I83223, I83229, I87031, I87311, I87312, I87313, I87319, I87331, I87332, I87333, I96, J340, K122, L00, L0100, L0101, L0103, L0109, L0201, L0202, L0211, L0212, L02211, L02212, L02213, L02214, L02215, L02219, L02221, L02222, L02223, L02224, L02229, L02232, L0231, L0232, L0233, L02411, L02412, L02413, L02414, L02415, L02416, L02419, L02511, L02512, L02611, L02612, L02619, L02811, L02818, L02821, L02828, L0291, L0292, L0293, L03011, L03012, L03019, L03031, L03032, L03039, L03041, L03042, L03111, L03112, L03113, L03114, L03115, L03116, L03119, L03125, L03126, L03129, L03211, L03213, L03221, L03311, L03312, L03313, L03314, L03315, L03316, L03317, L03319, L03811, L03818, L0390, L0391, L0501, L0591, L080, L081, L0889, L089, L120, L139, L2089, L250, L259, L280, L300, L303, L304, L308, L309, L438, L439, L599, L600, L601, L602, L603, L604, L605, L608, L609, L723, L728, L729, L732, L738, L7634, L84, L88, L89000, L89100, L89103, L89104, L89133, L89143, L89144, L89150, L89151, L89152, L89153, L89154, L89159, L89210, L89212, L89213, L89214, L89219, L89220, L89223, L89224, L89300, L89302, L89303, L89304, L89309, L89310, L89312, L89313, L89314, L89319, L89320, L89322, L89323, L89324, L89329, L8944, L89510, L89512, L89513, L89514, L89519, L89520, L89522, L89523, L89524, L89529, L89603, L89609, L89610, L89611, L89612, L89613, L89614, L89619, L89620, L89621, L89622, L89623, L89624, L89629, L89890, L89891, L89892, L89893, L89894, L89899, L8990, L8991, L8992, L8993, L8994, L8995, L900, L922, L923, L928, L929, L97112, L97122, L97209, L97211, L97212, L97213, L97219, L97221, L97222, L97223, L97225, L97229, L97302, L97309, L97311, L97312, L97313, L97314, L97315, L97318, L97319, L97321, L97322, L97323, L97324, L97325, L97329, L97401, L97402, L97409, L97411, L97412, L97413, L97414, L97415, L97416, L97418, L97419, L97421, L97422, L97423, L97424, L97425, L97426, L97429, L97501, L97502, L97503, L97504, L97509, L97511, L97512, L97513, L97514, L97515, L97516, L97518, L97519, L97521, L97522, L97523, L97524, L97525, L97526, L97528, L97529, L97801, L97802, L97809, L97811, L97812, L97813, L97814, L97815, L97818, L97819, L97821, L97822, L97823, L97824, L97825, L97828, L97829, L97901, L97902, L97909, L97911, L97912, L97913, L97915, L97919, L97921, L97922, L97923, L97924, L97928, L97929, L980, L98411, L98412, L98419, L98422, L98423, L98429, L98491, L98492, L98493, L98494, L98495, L98496, L98498, L98499, L988, L989, M726, N730, Q845,

© 2024 Hatfield KM et al. *JAMA Network Open.*

	R21, R238, S0006XA, S0086XA, S0096XA, S0100XA, S0100XD, S0120XA, S01301A, S0180XA, S0180XD, S1086XA, S1096XA, S1180XA, S1190XA, S20361A, S20362A, S20369A, S20461A, S20462A, S20469A, S2096XA, S21001A, S21001D, S21002A, S21109A, S21201A, S2190XA, S30860A, S30860D, S30861A, S30861D, S30863A, S31000D, S31104A, S31105D, S31105S, S31109D, S31109S, S3130XA, S31609A, S31809A, S31819A, S31829A, S31829D, S40261A, S40262A, S40861A, S40862A, S40869A, S41002A, S41101A, S41101D, S41102A, S50362A, S50861A, S50862A, S50869A, S51001A, S51001D, S51002A, S51801A, S51801D, S51802A, S51809A, S51811A, S60862A, S61401A, S61402A, S61402D, S70261A, S70262A, S70361A, S70362A, S70369A, S71001A, S71001D, S71002A, S71002D, S71101A, S71102A, S71102D, S80261A, S80262A, S80811A, S80812A, S80819A, S80821A, S80829A, S80861A, S80862A, S80869A, S81001A, S81001D, S81002A, S81801A, S81801D, S81801S, S81802A, S81802D, S81802S, S81809A, S81811A, S81811D, S81812A, S81812D, S81832A, S90211A, S90212A, S90421A, S90424A, S90425A, S90529A, S90562A, S90819A, S90821A, S90822A, S90829A, S90861A, S91001A, S91001D, S91002A, S91002D, S91002S, S91009A, S91101A, S91101D, S91102A, S91102D, S91104A, S91104D, S91105A, S91109A, S91201A, S91202A, S91205A, S91301A, S91301D, S91301S, S91302A, S91302D, S91309A, S91331A, S91332A, T8130XA, T8130XD, T8130XS, T8131XA, T8131XD, T8131XS, T8132XA, T8132XD, T8133XA, T8141XA, T8142XA, T8452XA, T86821 T8743, T8744, T8781, T8789, Z4800, Z4801, Z4802
Respiratory Infections	A310, A3790, B250, B342, B381, B390, B960, B9721, B9729, C3490, J00, J0100, J0101, J0110, J0111, J0120, J0130, J0140, J0141, J0180, J0181, J0190, J0191, J020, J028, J029, J0390, J040, J060, J069, J09X2, J101, J111, J1189, J1282, J1289, J129, J159, J168, J180, J181, J188, J189, J200, J208, J209, J219, J22, J300, J301, J302, J3089, J309, J310, J311, J312, J320, J321, J322, J323, J324, J328, J329, J330, J339, J343, J3481, J3489, J349, J3501, J370, J390, J392, J399, J40, J410, J411, J42, J439, J440, J441, J449, J4520, J4540, J4541, J45901, J45909, J45998, J470, J471, J479, J690, J80, J8410, J84112, J8489, J849, J90, J9600, J9601, J9610, J9611, J9620, J9621, J9690, J9801, J9809, J984, J988, J989, R042, R05, R051, R052, R053, R058, R059, R0600, R0602, R0603, R0609, R062, R0689, R069, R070, R071, R0781, R093, R0981, R0982, R918, U071, Z111, Z1152, Z1383, Z20822
Urogenital/Anogenital Infections	A539, A5400, A5402, A5403, A549, A5600, A5601, A5602, A5609, A5619, A562, A568, A5900, A5901, A5909, A599, A6000, A6004, A6009, A601, A609, A630, A638, A64, B009, B373, B3731, B3749, B977, C210, C519, C52, C539, D069, D071, D280, D398, F66, L292, L293, M542, N341, N342, N343, N485, N5312, N710, N711, N719, N72, N738, N739, N750, N751, N760, N761, N762, N763, N764, N765, N766, N7681, N7689, N770, N771, N816, N840, N841, N842, N848, N8500, N859, N870, N871, N879, N888, N889, N890, N891, N893, N894, N895, N898, N899, N900, N903, N904, N905, N907, N9089, N909, N920, N921, N926, N930, N938, N939, N9410, N9411, N9412, N9419, N942, N946, N94810 N94818 N94819 N9489, N949, N950, N952, N980, R102, R360, R361, R369, R875, R87610, R87611, R87612, R87613, R87615, R87616, R87618, R87619, R87620, R87622, R87628, R87629, R87810, R87811, R87820, R8789, R879, S30814A, S3140XA, S3141XA, T192XXA, T7421XA, T7421XD, Z01411, Z01419, Z0142, Z0441, Z113, Z1151, Z124, Z125,

© 2024 Hatfield KM et al. *JAMA Network Open.*

	Z1272, Z202, Z224, Z3009, Z30431, Z309, Z708, Z7251, Z7252, Z7253, Z87410, Z8742, Z91410
Urinary Tract Infections	A0682, B3741, N029, N10, N110, N130, N132, N136, N138, N139, N200, N201, N202, N209, N210, N218, N23, N2889, N289, N29, N3000, N3001, N3010, N3011, N3020, N3021, N3030, N3031, N3040, N3041, N3080, N3081, N3090, N3091, N311, N320, N323, N3281, N3289, N329, N33, N390, N3941, N3944, N398, N399, N401, N403, N410, N411, N413, N414, N4282, N5082, N8110, O2340, R300, R309, R310, R311, R312, R3121, R3129, R319, R32, R339, R350, R3589, R3911, R3915, R39198, R3981, R3982, R3989, R399, R823, R827, R8271, R8279, R828, R8281, R8289, R8290, R8299, R82998, T83511A, T83511D, T8613, Z436, Z466, Z87440, Z87442, Z87448

Note: International Classification of Diseases, Tenth Revision Clinical Modification (ICD-10-CM)

© 2024 Hatfield KM et al. *JAMA Network Open.*
