# Supplementary material for: Use of Multiplex Molecular Panels to Diagnose Urinary Tract Infection in Older Adults
Source: JAMA Netw Open. 2024 Nov 26;7(11):e2446842. doi: 10.1001/jamanetworkopen.2024.46842 (PMC11600226; doi:10.1001/jamanetworkopen.2024.46842)
Supplement: Supplement 2. — Data Sharing Statement [file jamanetwopen-e2446842-s002.pdf]

Data Sharing Statement

Hatfield. Use of Multiplex Molecular Panels to Diagnose Urinary Tract Infection in Older Adults. *JAMA Netw Open*. Published November 26, 2024. doi:10.1001/jamanetworkopen.2024.46842

Data
Data available: No

Additional Information
Explanation for why data not available: Data are available from CMS in the Virtual Research Data Center
